# Relationship between small dense low density lipoprotein and cardiovascular events in patients with acute coronary syndrome undergoing percutaneous coronary intervention

**DOI:** 10.1186/s12872-021-01979-7

**Published:** 2021-04-12

**Authors:** Jianwei Zhang, Lingjie He

**Affiliations:** 1 Department of Cardiology, Beijing Anzhen Hospital, Capital Medical University, Beijing Institute of Heart Lung and Blood Vessel Disease, Beijing Key Laboratory of Precision Medicine of Coronary Atherosclerotic Disease, Clinical Center for Coronary Heart Disease, Capital Medical University, Beijing, 100029 China; 2 Department of Outpatient, Beijing Friendship Hospital, Capital Medical University, Beijing, 100050 China

**Keywords:** Small dense LDL cholesterol, Acute coronary syndrome, Percutaneous coronary intervention, Risk assessment

## Abstract

**Background:**

Residual risk remained significant despite effective low density lipoprotein cholesterol (LDL-C) lowering treatment. Small dense low density lipoprotein cholesterol (sdLDL-C) as part of LDL-C has been found to be predictor of coronary heart disease (CHD) and cardiovascular (CV) events in patients with stable CHD independently of LDL-C. However, to date, few studies have explored the role of sdLDL-C in patients with acute coronary syndromes (ACS) undergoing percutaneous coronary intervention (PCI). Accordingly, this study aimed to evaluate the association of sdLDL-C with CV events in patients with ACS undergoing PCI.

**Methods:**

Patients hospitalized with ACS undergoing PCI were enrolled and followed up for 18 months. The risk of sdLDL-C for CV events was compared according to sdLDL-C quartiles. The primary outcome was major cardiovascular and cerebrovascular adverse events (MACCE), which was the composite of all cause of death, nonfatal myocardial infarction (MI), nonfatal stroke or unplanned repeat revascularization. A Cox proportional hazards regression model was performed to estimate the risk of CV events. Subgroup analysis according to diabetes status and LDL-C were performed separately for MACCE.

**Results:**

A total of 6092 patients were included in the analysis (age: 60.2 ± 10.13 years, male: 75.3%, BMI: 25.9 ± 3.33 kg/m^2^, dyslipidemia: 74.1% and diabetes: 44.5%). During 18 months of follow-up, 320 (5.2%) incident CV events occurred. Compared to the lowest sdLDL-C quartile group, patients in the highest quartile had a greater risk of CV events after multivariable adjustment (HR 1.92; 95% CI 1.37–2.70). In addition, it was mainly due to the increase of unplanned repeat revascularization. In the subgroup analyses, significant association was observed regardless of level of LDL-C and diabetes status.

**Conclusions:**

Patients with elevated sdLDL-C have a higher risk of CV events in Chinese patients with ACS undergoing PCI, providing additional value for better risk assessment.

**Supplementary Information:**

The online version contains supplementary material available at 10.1186/s12872-021-01979-7.

## Introduction

Death rates related to cardiovascular disease (CVD) have decreased, but it was still a leading cause of deaths as a result of aging, obesity and diabetes mellitus (DM) [1]. Dyslipidemia are widely recognized as a contributing risk factor for coronary heart disease (CHD) and stroke [2, 3]. However, residual risk remained significant despite effective low density lipoprotein cholesterol (LDL-C) lowering treatment in accordance with current guideline, including statin, ezetimibe and proprotein convertase subtilisin/kexin type 9 (PCSK9) antibodies [4–6]. In addition, LDL-C comprised of a heterogeneous lipoprotein particles including large, more buoyant LDL particles (lb‐LDL) and small dense low density lipoprotein (sdLDL) particles [7, 8], which could change independently from LDL-C [9]. Compared with lb‐LDL, small dense low density lipoprotein cholesterol (sdLDL-C) had higher ability for penetration into arterial wall, lower binding affinity for receptor, longer plasma half‐life and easier to oxidation [10]. Due to the atherogenic properties of sdLDL-C, using LDL‐C alone may underestimate actual risk in individuals when we evaluated cholesterol‐related CHD risk [11]. Therefore, risk assessment may be benefit from sdLDL-C measurement.

sdLDL-C has been found to be associated with increased risk for the development of CHD among the healthy participants with high or low risk of CVD [12, 13] and increased risk for cardiovascular (CV) events in patients with stable CVD [14]. However, to date, few studies have explored the role of sdLDL-C in patients with acute coronary syndromes (ACS) undergoing percutaneous coronary intervention (PCI). Accordingly, this study aimed to evaluate the association of sdLDL-C with CV events in patients with ACS undergoing PCI.

## Methods

### Study design and patients

In this cohort study, we consecutively included 9282 patients hospitalized for ACS and PCI from a top-ranked cardiovascular hospitals in China from January 2018 to December 2018. The main exclusion criteria were a body mass index (BMI) > 45 kg/m^2^, left ventricular ejection fraction (LVEF) < 30%, severe hepatic and renal insufficiency (estimated glomerular filtration rate [eGFR] < 30 ml/min), suspected familial hypertriglyceridemia (triglyceride ≥ 5.65 mmol/L), fibrate use, and malignancy diseases. We also excluded patients who underwent percutaneous coronary intervention through the femoral artery from this study. The retrospective cohort study was conducted in accordance with the Declaration of Helsinki and was approved by the Anzhen Hospital Institutional Ethical Review Board with a waiver of informed consent. Information related to the identity of the patient was concealed.

### Measurements

The data including patient demographics, smoking status, past medical history, laboratory results, PCI data, and medical treatments were collected from medical and nursing records. Blood samples were drawn after an overnight at least 8 h fasting. For patients with STEMI, blood samples were collected immediately on admission. Lipid profile were measured on the same day of collection. Fasting plasma glucose (FPG), glycosylated hemoglobin (HbA1c), Total cholesterol (TC) and triglyceride (TG) were measured by standard laboratory techniques. The measurement of sdLDL-C was performed in an automated homogeneous assay (Denka Seiken Co., Ltd., Tokyo, Japan) and analyzed on a Hitachi 7180 automatic analyzer [15].

### Treatment and procedure

All medication and operation were performed according to the guidelines [16]. All patients received aspirin and clopidogrel or ticagrelor prior to the procedure and 70–100 IU/kg unfractionated heparin intraoperative. A radial approach was used by 6 or 7 F guiding catheters. Second-generation drug eluting stents was implanted following appropriate predilation. The type of stent, fractional flow reserve (FFR), intravascular ultrasound (IVUS) and optical coherence tomography (OCT) were at the discretion of the interventionalist.

### Outcomes

All patients were followed up to incident primary outcome or for 18 months by telephone and only index events were included in the analysis. All events were recorded by two telephone records and inconsistent events were affirmed by a third record. Hospital records were also screened for clinical events. The primary outcome was major cardiovascular and cerebrovascular adverse events (MACCEs), which was the composite of all cause of death, nonfatal myocardial infarction (MI), nonfatal stroke or unplanned repeat revascularization. Death was defined as all causes of death regardless of cause of death [17]. Myocardial infarction was defined as the criteria for the fourth universal definition [18]. Stroke was adjudicated by the presence of as acute cerebral infarction established by the imaging or typical symptoms [19]. Unplanned repeat revascularization was defined as repeat PCI or surgical bypass of any segment of the target vessel or target lesion [17, 19]. Unstable angina was defined as rest, new-onset, or worsening angina without cardiac enzyme elevation [20]. Obesity was defined as BMI ≥ 28 kg/cm^2^. Hypertension was defined as a systolic blood pressure ≥ 140 mm Hg, diastolic blood pressure ≥ 90 mm Hg, or use of antihypertensive medications [21]. Diabetes mellitus was defined as taking hypoglycemic agents, a fasting (≥ 8 h) blood glucose of ≥ 7.0 mmol/L, or a nonfasting blood glucose of ≥ 11.10 mmol/L [22]. Dyslipidemia was defined as a fasting high-density lipoprotein cholesterol (HDL-C) < 40 mg/dL, TC > 200 mg/dL, LDL-C > 130 mg/dL, TG > 150 mg/dL or use of any lipid-lowering drug [23].

### Statistical analyses

Baseline patient characteristics were presented according to baseline sdLDL-C quartiles. Continuous variables were expressed as the mean ± standard deviation (SD) or median (interquartile range). The differences were estimated by one-way Analysis of Variance (ANOVA) for normal data or Kruskal–Wallis tests for non-normal distribution data followed by Bonferroni’s post hoc test. Categorical variables are expressed as numbers (percentage) and compared with a χ2 test or Fisher’s exact test.

Survival analyses were performed using Kaplan–Meier methods, log rank tests, and Cox proportional hazards regression models with backward stepwise selection methods according to baseline sdLDL-C quartiles. The following three models were adjusted for multivariate analysis: Model 1: age, gender, BMI; Model 2: model 1 + smoking status, hypertension, previous MI, previous stroke, syntax score, number of stents, total length of stents. Model 3: model 2 + TG, LDL-C, HDL-C, HbA1C, high sensitivity C-reactive protein, lipid-lowering medication use. Additionally, subgroups stratified according to diabetes status and dichotomized LDL-C and TG level (based on median value) were analyzed separately for cardiovascular events. Subgroup analyses were also conducted in each subgroup of age, sex, obesity, hypertension, previous MI, ACS type, hs-CRP. The heterogeneities in the relationship between subgroups were evaluated by adding multiplicative interaction terms in the multivariable models. In addition, we present the comparisons of baseline characteristics between participants who were eligible or not for the final analyses to test whether missing data would potential bias the results. All statistical analyses were performed using SPPS 24.0 software (IBM Corp., Armonk, NY, USA). A two-tailed value of *p* < 0.05 was required for statistical significance.

## Results

There were 9282 patients who met the inclusion criteria, of whom 3190 were excluded due to loss to follow-up (n = 781) or meet the major exclusion criteria (n = 2409). Finally, a total of 6092 patients were included in the analysis. Additional file 1: Figure S1 shows the patients’ flowchart. Comparison of baseline characteristics between participants who were eligible or not for the final analyses was displayed in Additional file 1: Table S1. Compared with the lost participants, eligible participants were significantly older. Though statistically significant, differences in BMI, current smoker and hypertension were not clinically relevant. Additionally, there was no statistically significant difference in lipid parameters.

### Baseline characteristics

The sdLDL-C had an approximately normal distribution with a mean of 28.2 ± 13.16 mg/dl (Additional file 1: Figure S2). Among high or low LDL-C (defined by the median of LDL-C) group of patients, wide variation in sdLDL-C was observed (Additional file 1: Figure S3a-b). With respect to diabetes status, significant differences of sdLDL-C were found (*p* = 0.002, Additional file 1: Figure S3c-d). Baseline characteristics data presented in Table 1. Among the included patients, there were 4586 (75.3%) male and mean ± SD age was 60.2 ± 10.13 years and BMI 25.9 ± 3.33 kg/m^2^. Diabetes and dyslipidemia was seen in 44.5% (2712) and 74.1% (4512) of patients, respectively. For the ACS type, 86.8% were unstable angina and the others were acute myocardial infarction (AMI). Among 2712 subjects with diabetes, 2171 were treated with oral hypoglycemic agents or insulin. Overall, almost all of patients were taking at least one prescription lipid-lowering medication and 98.4% were taking a statin with or without ezetimibe (20%). Of the analyzed coronary artery lesions, 16.9% were in left main artery, 59% were multivessel lesions, 15.2% were chronic total occlusion (CTO) lesion and the mean syntax score was 14 ± 7.49. FFR, IVUS and OCT were not widely practiced. The comparison of baseline characteristics according to sdLDL-C quartile are also shown in Table 1(Quartile 1: < 18.5 mg/dL, Quartile 2:18.5–25.5 mg/dL, Quartile 3: 25.5–35.1 mg/dL, Quartile 4: ≥ 35.1 mg/dL). There was statistically significant variation in age, gender, obesity, current smoking, hypertension, diabetes, dyslipidemia, hs-CRP and LVEF lipid parameters.

**Table 1:** Baseline characteristics of patients grouped by sdLDL-C quartile

sdLDL-C	Total	Quartile 1	Quartile 2	Quartile 3	Quartile 4	*p* value*
		< 18.5 mg/dL	18.5–25.5 mg/dL	25.5–35.1 mg/dL	≥ 35.1 mg/dL	
N (%)	6092 (100)	1593 (26.1)	1479 (24.3)	1516 (24.9)	1504 (24.7)	***–***
Age, y	60.2 ± 10.13	62.1 ± 9.9	60.7 ± 9.97	59.6 ± 9.71	58.2 ± 10.54	< 0.001
Male, n (%)	4586 (75.3)	1229 (77.2)	1140 (77.1)	1143 (75.4)	1074 (71.4)	0.001
BMI, kg/m^2^	25.9 ± 3.33	25.2 ± 3.27	25.8 ± 3.27	26.3 ± 3.38	26.4 ± 3.26	< 0.001
Obesity, n (%)	1397 (24.1)	284 (18.8)	327 (23.1)	403 (27.7)	383 (27.1)	< 0.001
Heart rate, bpm	72.1 ± 12.02	71.6 ± 11.29	71.9 ± 12.12	72.1 ± 12.03	73.1 ± 12.59	0.004
SBP, mmHg	128.2 ± 21.14	126.9 ± 20.8	128.3 ± 21.17	128.1 ± 21.22	129.6 ± 21.32	0.005
*Medical history and risk factors, n (%)*						
Current smoker	2200 (36.1)	509 (32)	549 (37.1)	542 (35.8)	600 (39.9)	< 0.001
Hypertension	3941 (64.7)	1029 (64.6)	914 (61.8)	1024 (67.5)	974 (64.8)	0.013
Diabetes	2712 (44.5)	692 (43.4)	642 (43.4)	658 (43.4)	720 (47.9)	0.028
Dyslipidaemia	4512 (74.1)	1123 (70.5)	1077 (72.8)	1118 (73.7)	1194 (79.4)	< 0.001
Previous MI	723 (11.9)	199 (12.5)	191 (12.9)	190 (12.5)	143 (9.5)	0.013
Previous Stroke	278 (4.6)	88 (5.5)	62 (4.2)	73 (4.8)	55 (3.7)	0.075
Previous PCI	1453 (23.9)	386 (24.2)	388 (26.2)	358 (23.6)	321 (21.3)	0.019
Previous CABG	153 (2.5)	48 (3)	36 (2.4)	37 (2.4)	32 (2.1)	0.456
*Laboratory tests*						
Cr, μmol/L	77.9 ± 51.62	82 ± 69.37	77.1 ± 46.53	77.6 ± 47.82	74.7 ± 35.16	0.001
eGFR, ml/min/1.73m^2^	123.6 ± 36.4	121.3 ± 32.87	123.3 ± 29.6	123.4 ± 29.68	126.5 ± 49.61	0.001
FBG, mmol/L	7 ± 2.54	6.8 ± 2.32	6.9 ± 2.44	7 ± 2.42	7.4 ± 2.9	< 0.001
HbA1C,%	6.6 ± 1.38	6.5 ± 1.25	6.6 ± 1.3	6.6 ± 1.31	6.8 ± 1.6	< 0.001
TC, mg/dL	158.8 ± 41.14	124.4 ± 22.06	145.1 ± 25.08	164 ± 26.81	203.5 ± 39.17	< 0.001
TG, mg/dL	123.2 (88.5–176.2)	85.1 (65.1–107.6)	109.4 (86.8–145.8)	142.8 (109.4–189.2)	180.5 (137.1–243.9)	< 0.001
HDL-C, mg/dL	41.4 ± 9.66	42.2 ± 10.16	41.3 ± 10.06	40.5 ± 9.44	41.5 ± 8.85	< 0.001
LDL-C, mg/dL	93.2 ± 34.31	65 ± 16.66	83.2 ± 20.12	97.4 ± 23.63	128.8 ± 35.87	< 0.001
hs-CRP, mg/L	1.35 (0.6–3.4)	1.0 (0.4–2.7)	1.2 (0.5–3.2)	1.5 (0.6–3.4)	1.8 (0.8–4.2)	< 0.001
TNI, μg/L	0.9 ± 5.34	0.7 ± 4.87	0.5 ± 3.42	0.9 ± 5.46	1.5 ± 6.99	< 0.001
LVEF,%	61.2 ± 7.74	60.8 ± 7.65	61 ± 7.98	61.4 ± 7.61	61.6 ± 7.69	0.302
*ACS type, n (%)*						
Unstable angina	5286 (86.8)	1410 (88.5)	1303 (88.1)	1303 (85.9)	1270 (84.4)	0.002
AMI	806 (13.2)	183 (11.5)	176 (11.9)	213 (14.1)	234 (15.6)	-
*Medication on admission, n (%)*						
Aspirin	4944 (81.2)	1298 (81.5)	1229 (83.1)	1215 (80.1)	1202 (79.9)	0.100
clopidogrel	3159 (52.3)	797 (50.5)	803 (54.7)	781 (52)	778 (52)	0.139
ticagrelor	918 (15.2)	231 (14.6)	209 (14.2)	244 (16.2)	234 (15.7)	0.392
statin	5062 (83.1)	1289 (80.9)	1220 (82.5)	1266 (83.5)	1287 (85.6)	0.006
Ezetimibe	668 (11)	147 (9.2)	151 (10.2)	168 (11.1)	202 (13.4)	0.002
Any antidiabetic agents	1285 (21.1)	344 (21.6)	298 (20.1)	301 (19.9)	342 (22.7)	0.181
*Medication at discharge, n (%)*						
Aspirin	5956 (97.8)	1555 (97.6)	1451 (98.1)	1482 (97.8)	1468 (97.6)	0.77
clopidogrel	4289 (70.4)	1130 (70.9)	1059 (71.6)	1027 (67.7)	1073 (71.3)	0.071
ticagrelor	1803 (29.6)	463 (29.1)	420 (28.4)	489 (32.3)	431 (28.7)	0.071
ACEI/ARB	2655 (43.6)	673 (42.2)	652 (44.1)	688 (45.4)	642 (42.7)	0.284
β-Blocker	3926 (64.4)	1034 (64.9)	977 (66.1)	959 (63.3)	956 (63.6)	0.352
statin	5996 (98.4)	1567 (98.4)	1455 (98.4)	1492 (98.4)	1482 (98.5)	0.981
Ezetimibe	1219 (20)	259 (16.3)	255 (17.2)	331 (21.8)	374 (24.9)	< 0.001
Any antidiabetic agents	2171 (35.6)	562 (35.3)	509 (34.4)	516 (34)	584 (38.8)	0.024
*Angiographic Coronary anatomy, n (%)*						
Any left main disease	1029 (16.9)	262 (16.4)	223 (15.1)	273 (18)	271 (18)	0.094
Multivessel disease	3597 (59)	885 (55.6)	818 (55.3)	916 (60.4)	978 (65)	< 0.001
Others	2290 (37.6)	648 (40.7)	619 (41.9)	546 (36)	477 (31.7)	< 0.001
CTO	928 (15.2)	233 (14.6)	253 (17.1)	218 (14.4)	224 (14.9)	0.141
Lesions > 20 mm	3703 (60.8)	955 (59.9)	844 (57.1)	918 (60.6)	986 (65.6)	< 0.001
Syntax	14 ± 7.49	14.1 ± 7.39	13.5 ± 6.97	14.5 ± 7.97	14 ± 7.55	0.04
*Treated vessel, n (%)*						
LM	623 (10.2)	164 (10.3)	138 (9.3)	166 (10.9)	155 (10.3)	0.537
LAD	3143 (51.6)	833 (52.3)	748 (50.6)	775 (51.1)	787 (52.3)	0.709
LCX	1763 (28.9)	425 (26.7)	423 (28.6)	454 (29.9)	461 (30.7)	0.074
RCA	2436 (40.0)	647 (40.6)	585 (39.6)	614 (40.5)	590 (39.2)	0.824
DCB	375 (6.2)	110 (6.9)	83 (5.6)	89 (5.9)	93 (6.2)	0.471
FFR	31 (0.5)	14 (0.9)	5 (0.3)	6 (0.4)	6 (0.4)	0.117
IVUS	137 (2.2)	41 (2.6)	44 (3.0)	35 (2.3)	17 (1.1)	0.005
OCT	108 (1.8)	31 (1.9)	23 (1.6)	27 (1.8)	27 (1.8)	0.877
Number of stents	1.7 ± 0.82	1.7 ± 0.82	1.6 ± 0.8	1.7 ± 0.84	1.7 ± 0.82	0.068
Total length of stents, mm	39.9 ± 24.5	39.1 ± 25.56	38.8 ± 23.24	41.1 ± 24.7	40.4 ± 24.52	0.323

Values are mean ± SD, median (interquartile range), or n (%). *p value for test of difference group of participants according to sdLDL-C quartile by one-way Analysis of Variance or χ^2^ test. BMI body mass index, SBP systolic blood pressure, MI myocardial infarction, PCI percutaneous coronary intervention, CABG Coronary Artery Bypass Grafting, Cr creatinine, UA Uric Acid, eGFR estimated glomerular filtration rate, FPG fasting plasma glucose, HbA1C Glycosylated haemoglobin, TC total cholesterol, TG triglyceride, HDL-C high-density lipoprotein-cholesterol, LDL-C low-density lipoprotein-cholesterol, sdLDL-C, Small dense low-density lipoprotein cholesterol, LVEF left ventricular ejection fraction, AMI acute myocardial infarction, ACEI angiotensin converting enzyme inhibitor, ARB angiotensin II receptor blocker, CTO chronic total occlusion, LM left-main artery, LAD left anterior descending artery, LCX left circumflex artery,RCA right coronary artery, DCB drug-coated balloon, FFR Fractional Flow Reserve, IVUS intravascular ultrasound, OCT optical coherence tomography

### Relationship sdLDL-C of with cardiovascular events

**Fig. 1:**
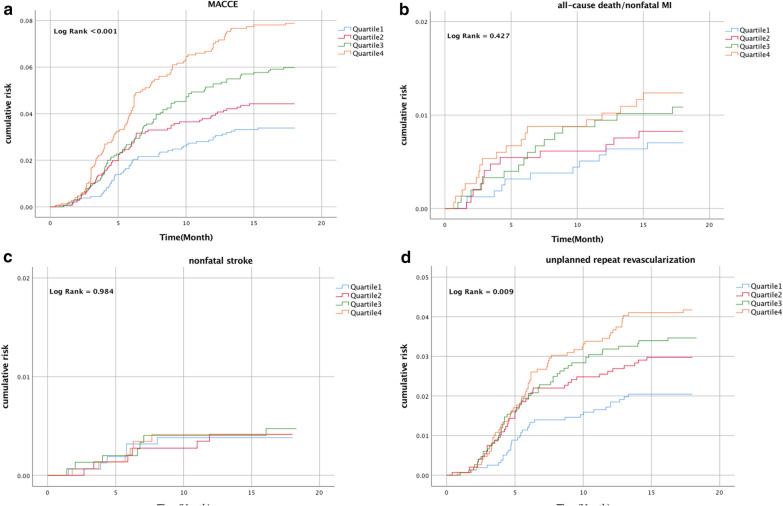
Kaplan–Meier curve according to quartile of sdLDL-C for MACCE (**a**), all-cause death/nonfatal MI (**b**), nonfatal stroke (**c**) and unplanned repeat revascularization (**d**). sdLDL-C small dense low-density lipoprotein cholesterol, MACCE major adverse cardiac and cerebrovascular events, MI myocardial infarction

A total of 320 (5.2%) incident MACCE occurred during 18 months of follow-up. Table 2 shown that the increase of MACCE in the high quartile of sdLDL-C group was mainly due to the increase of unplanned repeat revascularization, which also indicated by the Kaplan–Meier curve according to quartile of sdLDL-C (Fig. 1). Hazard ratios for incidence of CV events by quartile of sdLDL-C are presented in Table 3. Patients are grouped in sdLDL-C quartiles, with Quartile 1 having the lowest and Quartile 4 the highest risk of CV events after adjusted with model 3 (hazard ratio [HR] 1.71, 95% confidence interval [CI] 1.21–2.41, p for trend = 0.015). In subgroup analyses according to diabetes status (Table 4), the difference of HR between the extreme quartile of sdLDL-C remained statistically significant in model 3 (non-diabetes: HR 2.98, 95% CI 1.28–6.95; diabetes: HR 2.30, 95% CI 1.45–3.67). And there was no statistically significant interaction between sdLDL-C and diabetes status (*P* for interaction = 0.789). Using group of patients with lowest quartile of sdLDL-C and LDL-C < 55md/dL as a reference (Table 4), patients in the high sdLDL-C had a greater risk of CV events after adjusted with model 3 (Q2 vs. Q1: HR 0.41, 95% CI 0.08–2.10; Q3 vs. Q1: HR 1.49, 95% CI 0.47–4.68; Q4 vs. Q1: HR 3.94, 95% CI 1.46–10.60; *P* for interaction = 0.494). Similar results were obtained in a group of patients with LDL-C ≥ 55md/dL.

**Table 2:** Incident of cardiovascular events according to quartile of sdLDL-C

	Quartile 1	Quartile 2	Quartile 3	Quartile 4
MACCE	53 (3.3)	64 (4.3)	89 (5.9)	114 (7.6)
All cause death	5 (0.3)	4 (0.3)	9 (0.6)	7 (0.5)
Non-fatal myocardial infarction	6 (0.4)	9 (0.6)	8 (0.5)	11 (0.7)
Non-fatal stroke	6 (0.4)	6 (0.4)	7 (0.5)	6 (0.4)
Unplanned repeat revascularization	32 (2.0)	43 (2.9)	51 (3.4)	60 (4.0)

Values expressed are n (%). Quartile 1: < 18.5 mg/dL, Quartile 2:18.5–25.5 mg/dL, Quartile 3: 25.5–35.1 mg/dL, Quartile 4: ≥ 35.1 mg/dL. MACCE major adverse cardiac and cerebrovascular event

**Table 3:** Risk of cardiovascular events according to quartile of sdLDL-C

	Model 1*	Model 2*	Model 3*
Quartile 1 (reference)	–	–	–
Quartile 2	1.31 (0.91–1.89)	1.28 (0.88–1.87)	1.22 (0.87–1.79)
Quartile 3	1.77 (1.26–2.48)	1.65 (1.16–2.35)	1.39 (0.97–1.98)
Quartile 4	2.33 (1.68–3.23)	2.21 (1.57–3.09)	1.71 (1.21–2.41)
P for trend	< 0.001	< 0.001	0.015

Values are hazard ratio (95% confidence interval) unless otherwise indicated. Quartile 1: < 18.5 mg/dL, Quartile 2:18.5–25.5 mg/dL, Quartile 3: 25.5–35.1 mg/dL, Quartile 4: ≥ 35.1 mg/dL. * Model 1: age, gender, BMI; Model 2: model 1 + smoking status, hypertension, previous MI, previous stroke, syntax score, number of stents, total length of stents. Model 3: model 2 + TG, LDL-C, HDL-C, HbA1C, high sensitivity C-reactive protein, lipid-lowering medication use. Hazard ratios and 95% confidence intervals were obtained from Cox proportional hazards models

**Table 4:** Hazard ratios for cardiovascular events according to sdLDL-C quartile and different LDL-C level and diabetes status

	Quartile 1	Quartile 2	Quartile 3	Quartile 4	p for trend
		HR (95% CI)			
Non-diabetes	1 (reference)	0.963 (0.34–2.75)	1.91 (0.79–4.6)	2.98 (1.28–6.95)*	0.014
Diabetes	1 (reference)	1.48 (0.89–2.46)	1.49 (0.90–2.47)	2.30 (1.45–3.67)*	0.004
LDL-C < 55 mg/dL	1 (reference)	0.41 (0.08–2.10)	1.49 (0.47–4.68)	3.94 (1.46–10.60)*	0.001
LDL-C ≥ 55 mg/dL	1 (reference)	1.15 (0.78–1.69)	1.33 (1.86–2.64)*	2.1 (1.56–3.09)*	< 0.001

Values expressed are hazard ratio (95% confidence interval). sdLDL-C small dense low-density lipoprotein cholesterol, LDL-C low-density lipoprotein cholesterol, HR hazard ratio, CI indicates confidence interval. *p < 0.05. Adjusted for age, gender, BMI, smoking status, hypertension, previous MI, previous stroke, syntax score, number of stents, total length of stents, TG, LDL-C, HDL-C, HbA1C, high sensitivity C-reactive protein, lipid-lowering medication use

**Fig. 2:**
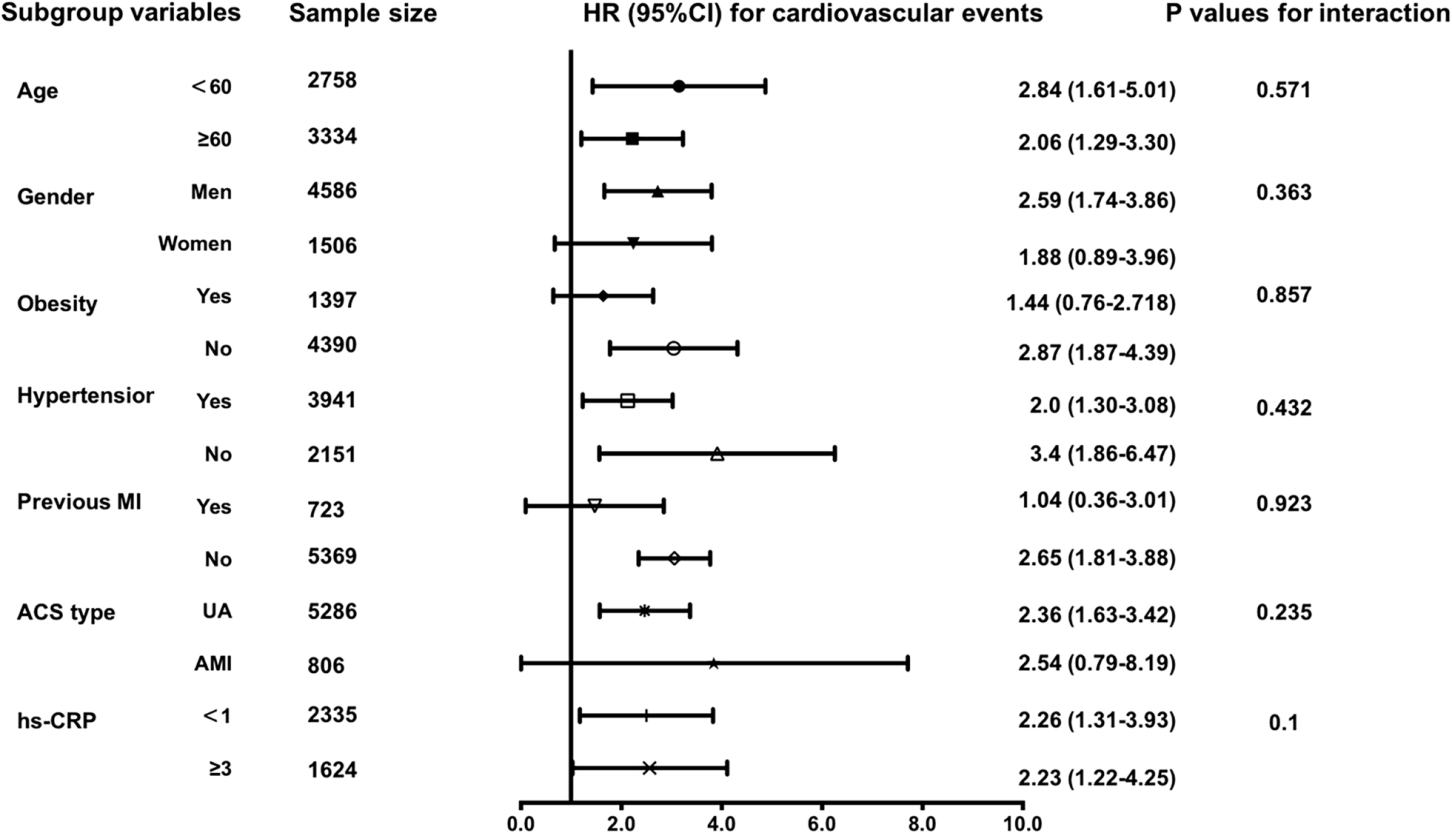
Extreme quartile adjusted hazard ratios for associations with cardiovascular events in subgroups of cardiovascular risk factors. MI myocardial infarction, ACS acute coronary syndrome, sdLDL-C Small dense low-density lipoprotein cholesterol. *p < 0.05. Adjusted for age, gender, BMI, smoking status, hypertension, previous MI, previous stroke, syntax score, number of stents, total length of stents, TG, LDL-C, HDL-C, HbA1C, high sensitivity C-reactive protein, lipid-lowering medication use excluding subgroup variables

Finally, stratified analysis by age, sex, obesity, hypertension, previous MI, ACS type, hs-CRP was conducted as shown in Fig. 2. The multivariable-adjusted risk for CV events tended to be higher in subjects with highest quartile of sdLDL-C than in those with lowest quartile of sdLDL-C with or without statistically significant in all of subgroups analyzed in model 3. No significant interaction between sdLDL-C and these subgroups was observed. (all *P* values for interaction > 0.05).

## Discussion

In this study, we report that patients with a high sdLDL-C were more likely to be have a high risk of CV events in Chinese patients with ACS undergoing PCI. This higher risk remained significant in patients regardless of diabetes status, LDL-C and TG levels. To our knowledge, our study is the first large-scale trial estimating the association between sdLDL-C and the risk of CV events in patients with ACS undergoing PCI. Actually, there may be no significant increase in LDL-C levels in some patients with diabetes or metabolic syndrome [24]. Therefore, it is clinically valuable to measure sdLDL-C for estimating the risk of CV events in patients with ACS undergoing PCI for its highly atherogenic properties. Overall, sd-LDL-C was favorable for distinguishing patients with high risk of CV events beyond LDL-C level.

There are several observational studies reported the associations between sdLDL-C and subclinical atherosclerosis [13, 25, 26] or CAD [11, 12, 27]. In a small prospective study, an increase of sdLDL-C was shown to predict intima media thickness (IMT) and insulin resistance [28]. Results from a trial with 816 patients without diabetes or CVD showed sdLDL-C can independently predict arterial stiffness progression [29]. Moreover, several studies have indicated that sdLDL-C was independently associated with the progression of carotid atherosclerosis [13, 25, 26]. The Suita Study followed 2,034 general urban Japanese residents for an average of 11 years and have suggested that the highest quartile of sdLDL-C level was associsated with a 3.3-fold higher risk of incident CHD compared with the lowest quartile (95% CI 1.3‒8.2) [30]. The Multi-Ethnic Study of Atherosclerosis, which included 4387 USA patients and followed up for an average of 8.5 year, demonstrated that adjusted hazard ratios for incident CHD between extreme quartile of sdLDL-C was 2.4 [11]. The ARIC study, which included 11,419 patients and followed up for 11-year, demonstrated that sdLDL-C was associated with incident CHD [31]. And the association remained significant regardless of LDL-C levels in these studies. Meanwhile, Duran EK et al. also indicated that sdLDL-C affected atherogenesis independently of LDL-C and hs-CRP [32], which was consistent with results of the above mentioned research. Furthermore, sdLDL-C predicted the CHD risk not only in patients at high cardiovascular risk [12], but also at low cardiovascular risk according to LDL-C values [31], therefore providing additional value for better risk assessment.

In addition, several studies reported the association between sdLDL-C and coronary stenosis severity or prognosis in patients with CAD. Koba S et al. recruited 482 stable CHD patients and 389 patients without CHD and indicated sdLDL-C level was more efficacious in predicting coronary severity [33]. A cohort study from china suggested that increased sdLDL-C were associated with higher risk of CV events in patients with diabetes and stable CAD [14]. Therefore, the current study might provide valuable further information on the relationship of sdLDL-C and CV events in patients with ACS undergoing PCI.

Also, several studies have shown sdLDL-C was closely related to stroke [26]. A cross-sectional study included a total of 754 acute ischemic stroke (AIS) patients indicated that sdLDL-C levels was an independent predictor of NIHSS scores and the severity of cerebral artery calcification [34]. A study enrolled 530 elderly patients hospitalized within 48 h after stroke suggested high sdLDL-C were associated with a greater risk for ischemic stroke [35]. Another study recruited 355 AIS and 171 non-AIS patients and found that elevated sdLDL-C was associated with a higher incidence of AIS [36].

In this study, we also report that sdLDL-C was associations with increased risk of CV events regardless of diabetes status, which seems to be not very consistent with previous study. The Multi-Ethnic Study of Atherosclerosis demonstrated that elevated sdLDL-C was an independent risk factors for CHD only in non-diabetic patients but not in diabetes patients [11]. A cohort study from china indicated that elevated sdLDL-C was associated with greater risk of CV events in DM patients with stable CAD but not non-diabetic patients [14]. There were several limits for these studies. First, TG had a strong relationship with sdLDL-C [9], which means the inclusion of both variables in the final multivariable regression model may bias the results. Second, the small sample size in the subgroup may explain the overall positive results but negative in subgroup analysis in these studies. Therefore, the negative result should not be used as a definite conclusion. Actually diabetic and non-diabetic patients accounted for almost half of the patients in the final analysis in our study, which means that our research may come up with positive results. In another study, the relationship between sdLDL-C and CHD was significant regardless of diabetes ststus, which was consistent with our study [12]. Moreover, patients with DM was likely to have smaller LDL. sdLDL-C had strong association with metabolic syndrome [37]. insulin resistance [9] and subclinical diabetes status [38].

As the size of LDL particles changes from the largest to the smallest, the relative contents of cholesterol and phospholipids are significantly reduced, while TG and protein are significantly increased. The significant conformational changes in apolipoprotein B‐100 on the surface of LDL particles leaded to decrease in LDL receptor affinity, plasma clearance and antioxidant capacity [39]. Previous study confirmed that most of sdLDL apolipoprotein B‐100 was derived from the transformation of lbLDL or even directly from VLDL apolipoprotein B‐100. Moreover, sdLDL apolipoprotein B-100 stayed much longer in plasma (74 h) than lbLDL apolipoprotein B-100 (40 h) [40]. Ikezaki et al. indicated that small dense LDL was the most atherogenic lipoprotein parameter for its longer plasma residence time and smaller size [41]. These properties gave sdLDL-C more time for desialylation, glycation and oxidation, made it easier to penetrate endothelial cells and easily be swallowed by macrophages through modified LDL receptors [31]. Krychtiuk et al. showed that sdLDL-C was associated with an increase of non-classical monocytes (NCM; CD14 + CD16 + +) and a decrease of classical monocytes (CM; CD14 +  + CD16-) [42]. In a prospective study within the Women’s Health Study, sdLDL-C was a strong risk factor for MI but not peripheral artery disease (PAD), which indicated that elevated sdLDL-C may be relevant to instability and vulnerability plaque rather than the more stable plaque [32].

This study has several limitations to consider. First, our results may be affected by residual confounding in this observational study. Second, there may be a significant decrease in sdDL-C in patients with AMI, but our study still found a close relationship between preoperative sdLDL-C concentration and prognosis. In addition, we collected blood samples immediate after admission to reducing the effect on sdLDL-C. Third, time-dependent analysis was not available for only once measurement of sdLDL-C at baseline. Fourth, unable to obtain follow-up information of medical treatments may bias the results. Finally, current research findings may not be generalizable to other ethnic groups because the participants in our study were only Chinese. Therefore, our findings should be confirmed in other ethnic populations.

## Conclusions

Among Chinese patients with ACS undergoing PCI, patients with high sdLDL-C were at a higher risk of developing CV. These findings may help identify high-risk patients with cardiovascular events beyond LDL-C and those patients may benefit from more aggressive therapy.

## Supplementary Information

**Additional file 1: Figure S1**. Flowchart. **Table S1**. Characteristics of the lost participants and eligible participants. **Figure S2**. Distribution of small dense low density lipoprotein cholesterol. **Figure S3**. Distribution of small dense low density lipoprotein cholesterol in groups of patients according to low density lipoprotein cholesterol level and diabetes status.

## Data Availability

All data generated or analysed during this study are included in this published article and its supplementary information files.

## Abbreviations

BMI: Body mass index
FPG: Fasting plasma glucose
HbA1C: Glycosylated hemoglobin
TC: Total cholesterol
HDL-C: High-density lipoprotein-cholesterol
LDL-C: Low-density lipoprotein-cholesterol
sdLDL-C: Small dense low-density lipoprotein cholesterol
ACS: Acute coronary syndrome
AMI: Acute myocardial infarction
ACEI: Angiotensin converting enzyme inhibitor
ARB: Angiotensin II receptor blocker
PCI: Percutaneous coronary intervention
CTO: Chronic total occlusion
LM: Left main artery
LAD: Left anterior descending artery
LCX: Left circumflex artery
RCA: Right coronary artery
FFR: Fractional Flow Reserve
IVUS: Intravascular ultrasound
OCT: Optical coherence tomography
HR: Hazard ratio
CI: Confidence interval
