# Supplementary material for: Relationship between small dense low density lipoprotein and cardiovascular events in patients with acute coronary syndrome undergoing percutaneous coronary intervention
Source: BMC Cardiovasc Disord. 2021 Apr 12;21:169. doi: 10.1186/s12872-021-01979-7 (PMC8040195; doi:10.1186/s12872-021-01979-7)
Supplement: Supplementary file 1 — Additional file 1: Figure S1. Flowchart. Table S1. Characteristics of the lost participants and eligible participants. Figure S2. Distribution of small dense low density lipoprotein cholesterol. Figure S3. Distribution of small dense low density lipoprotein cholesterol in groups of patients according to low density lipoprotein cholesterol level and diabetes status. [file 12872_2021_1979_MOESM1_ESM.docx]

**Relationship between Small dense low density lipoprotein and Cardiovascular Events in Patients with Acute Coronary Syndrome Undergoing Percutaneous Coronary Intervention**

Jianwei Zhang1, Lingjie He 2

1Department of Cardiology, Beijing Anzhen Hospital, Capital Medical University, Beijing Institute of Heart Lung and Blood Vessel Disease, Beijing Key Laboratory of Precision Medicine of Coronary Atherosclerotic Disease, Clinical center for coronary heart disease, Capital Medical University，Beijing 100029, China

2Department of Outpatient, Beijing Friendship Hospital, Capital Medical University, Beijing, 100050, China

Correspondence to: Dr. Ling-jie He, Department of Outpatient, Beijing Friendship Hospital, Capital Medical University, Beijing, 100050, China (Tel: 86-10-50849069. Fax: 86-10-50849069. Email: hlj925@sohu.com

Email addresses: Lingjie He: hlj925@sohu.com


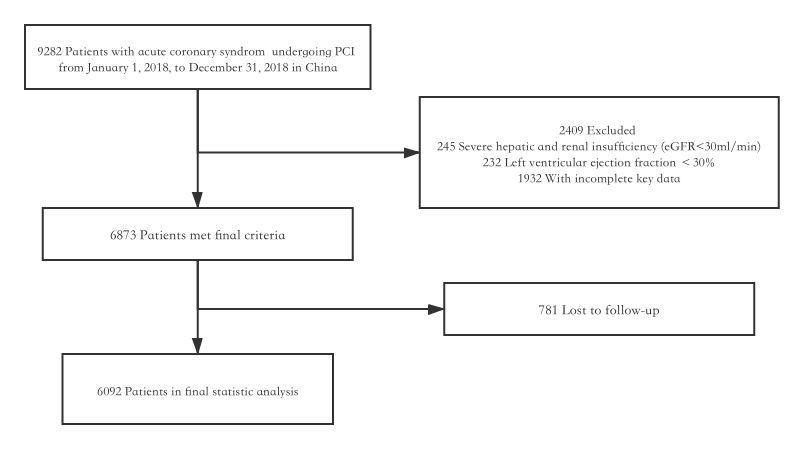
 Figure S1. flowchart

Table S1. Characteristics of the lost participants and eligible participants

|  | Lost participants | Eligible participants | *P value** |
| --- | --- | --- | --- |
| N | 3190 | 6092 | - |
| Age, y | 59.4±9.86 | 60.2±10.13 | ＜0.001 |
| Male,n (%) | 2410(75.5%) | 4586(75.3) | 0.839 |
| BMI, kg/m2 | 26.2±3.17 | 25.9±3.32 | 0.001 |
| SBP, mmHg | 129.2±20.31 | 128.2±21.14 | 0.024 |
| Medical history and risk factors, n (%) | | |  |
| Current smoker | 1297(40.6) | 2200(36.1) | ＜0.001 |
| Hypertension | 2163(67.7) | 3941(64.7) | 0.003 |
| Diabetes | 1362(42.7) | 2712(44.5) | 0.09 |
| FPG, mmol/L | 7.1±3.54 | 7.0±2.54 | 0.064 |
| HbA1C,% | 6.6±1.43 | 6.6±1.38 | 0.789 |
| TC | 160.4±41.15 | 158.8±41.14 | 0.081 |
| TG | 154.7±124.34 | 150.0±109.97 | 0.061 |
| HDL-C | 41.8±10.08 | 41.4±9.66 | 0.091 |
| LDL-C | 93.9±33.47 | 93.2±34.31 | 0.39 |
| hs-CRP | 3.1±4.43 | 3.1±4.44 | 0.006 |

Values are mean± SD, median (interquartile range), or n (%). *p value for test of difference across the lost participants and eligible participants by Student’s t test or the χ2 test. BMI body mass index, SBP systolic blood pressure, FPG fasting plasma glucose, HbA1C Glycosylated haemoglobin, TC total cholesterol, TG triglyceride, HDL-C high-density lipoprotein-cholesterol, LDL-C low-density lipoprotein-cholesterol, hs-CRP high sensitivity C-reactive protein


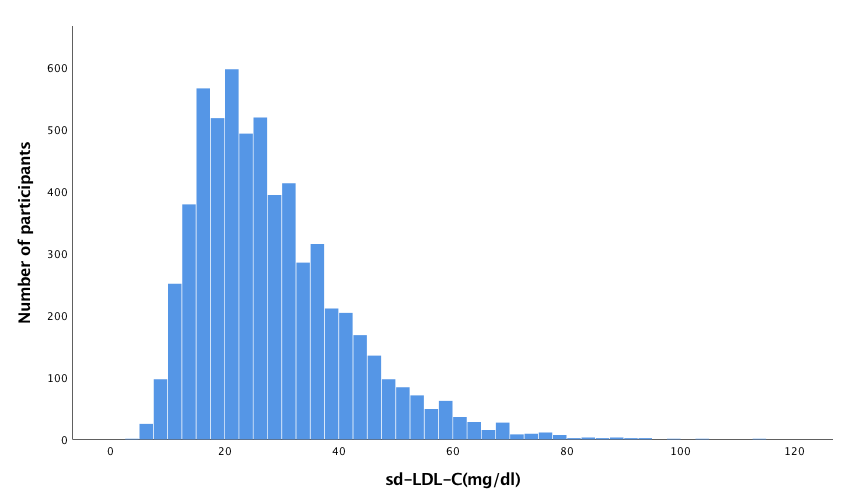


Figure S2. Distribution of small dense low density lipoprotein cholesterol


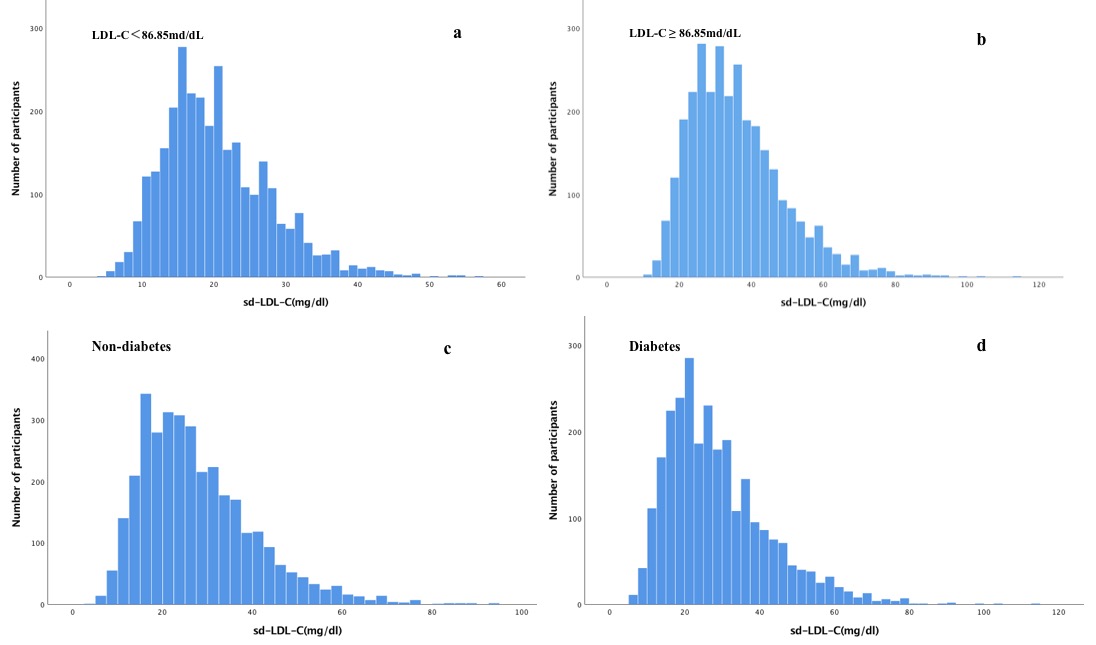


Figure S3. Distribution of small dense low density lipoprotein cholesterol in groups of patients according to low density lipoprotein cholesterol level and diabetes status
